# Reviewer acknowledgement 2013

**DOI:** 10.1186/1471-2407-14-50

**Published:** 2014-02-03

**Authors:** Dafne Solera

**Affiliations:** 1 BioMed Central, 236 Gray’s Inn, Road, London WC1X 8HB, UK

## Abstract

**Contributing reviewers:**

The editors of *BMC Cancer* would like to thank all our reviewers who have contributed to the journal in Volume 13 (2013).

## 

Miguel Abal

Spain

Jean-Pierre Abastado

Singapore

Ciro Abbondanza

Italy

Daniel Abbott

USA

Ahmed Abdelaziz

Egypt

Sherif Abdel-Misih

USA

Omar Abdel-Wahab

USA

Ahmed Abdulamir

Malaysia

Michael Abend

Germany

Filippo Acconcia

Italy

Stephen Ackland

Australia

Liana Adam

USA

Foluso Ademuyiwa

USA

Pavan Adiseshaiah

USA

Yaw Adjepong

USA

Ilir Agalliu

USA

Jai Prakash Agarwal

India

Dinesh Kumar Ahirwar

USA

Imran Ahmad

UK

Hojjat Ahmadzadehfar

Germany

Yong Chan Ahn

South Korea

Helmut Ahrens

Germany

Ayal Aizer

USA

Sadako Akashi-Tanaka

Japan

Hideyuki Akaza

Japan

Mohammad Reza Akbari

Canada

Andre Albergaria

Portugal

Scott Albert

USA

Mohammed Aleskandarany

UK

Elizabeth Algar

Australia

Yazeed Alhiyari

USA

Malcolm Alison

UK

James Allan

UK

Eugenia Allegra

Italy

Margaret Allman-Farinelli

Australia

Fabio Almeida

Brazil

Martin Almquist

Sweden

Fahd Al-Mulla

Kuwait

Redhwan Al-Naggar

Malaysia

Sergio Alonso

Spain

Gianfranco Alpini

USA

Rachel Altura

USA

Robert Amato

USA

Bhasker Amatya

Australia

Maurizio Amichetti

Italy

Ali Amin

UK

Martine Amiot

France

She-Juan An

China

Ioannis Anagnostopoulos

Germany

Satoshi Anai

Japan

Daniel Anaya

USA

Ladislav Andera

Czech Republic

M Robyn Andersen

USA

Annie Anderson

UK

Endre Anderssen

USA

Patiyan Andersson

Australia

Nicolaus Andratschke

Germany

Ove Andrén

Sweden

Gabriella Andreotti

USA

Mei-Kim Ang

Singapore

Valentina Angeloni

Italy

Vladimir Anisimov

Russian Federation

Jose Leopoldo Ferreira Antunes

Brazil

Luis Antunes

Portugal

Hajer Aounallah-Skhiri

Tunisia

Valentina Appierto

Italy

Udayan Apte

USA

Jeanny Aragon-Ching

USA

Michael Arand

Switzerland

Vera Araujo

Brazil

Marc Arbyn

Belgium

Takaaki Arigami

Japan

Aziz Aris

Canada

Jo Armes

UK

Lauren Arnold

USA

Juan Ignacio Arraras

Spain

Amanda Arrington

USA

Thiruvengadam Arumugam

USA

Mariko Asai

Japan

Hassan Ashktorab

USA

Jonathan Ashman

USA

Maurice Efana Asuquo

Nigeria

Dagfinn Aune

UK

Stefanie Aust

Austria

Romane Auvergne

USA

Eeva Auvinen

Finland

Amir Avan

Netherlands

Murugan Avaniyapuram Kannan

USA

Çigir Avci

Turkey

Claudio Avellini

Italy

Christophe Avenel

Sweden

Matias Avila

Spain

Ahmad Awada

Belgium

Niranjan Awasthi

USA

Olga Azarenko

USA

Asfar Azmi

USA

Maria Azrad

USA

Hideo Baba

Japan

Max Bachem

Germany

Michael Bachmann

Germany

Ahmed Badawy

Egypt

Bruce Baguley

New Zealand

Ruqia Baig

Pakistan

Gian Luca Baiocchi

Italy

Otavio Baiocchi

Brazil

Ana M Bajo

Spain

Petros Bakakos

Greece

Samuel Bakhoum

USA

Kyriaki Bakirtzi

USA

Amanjit Bal

India

Shashi Bala

USA

Francesc Balaguer

Spain

Claudia Baldus

Germany

Amy Baldwin

Grenada

Fatima Baltazar

Portugal

Vimla Band

USA

Pratiti Bandopadhayay

USA

Partha Banerjee

USA

Bo-Ying Bao

Taiwan

Voichita Bar Ad

USA

Dario Baratti

Italy

Federica Barbieri

Italy

Marco Barchi

Italy

Domingo Barettino

Spain

Debmalya Barh

India

Daniela Barilà

Italy

Sophie Barillé-Nion

France

David Barker

USA

Joyce Barlin

USA

Benjamin Barré

France

Andrea Barrio

USA

Kathryn Barry

USA

John Bartlett

Canada

Elisabeth Barton

USA

John Basile

USA

Konrad Basler

Switzerland

Nuray Bassullu

Turkey

Nermine Basta

UK

Devraj Basu

USA

Tone Bathen

Norway

Ayse Batova

USA

Jyotsna Batra

Australia

Michael Baudis

Switzerland

Iacopo Baussano

France

Heather Becker

USA

Therese Becker

Australia

Laurent Bedenne

France

Vanessa Lea Beesley

Australia

Louk Beex

Netherlands

Ruud Bekkers

Netherlands

Antonino Belfiore

Italy

Abbes Belkhiri

USA

Joaquim Bellmunt

USA

Irit Ben-Aharon

Israel

Abdulbari Bener

Qatar

David Bentrem

USA

Carmen Berasain

Spain

Michael Berens

USA

Anke Bergmann

Brazil

Elke Bergmann-Leitner

USA

Martin Bergo

Sweden

Dominique Bernard-Gallon

France

Sonja Berndt

USA

Maria Grazia Bernengo

Italy

Wolfram Bernstorff

Germany

Franco Berrino

Italy

Lev Berstein

Russian Federation

Lucio Bertario

Italy

Julien Berthiller

France

Andrea Bertotti

Italy

Fred Bertrand

USA

Kimberly Bertrand

USA

Marianne Berwick

USA

Kishor Bhakat

USA

Vishakha Bhave

USA

Xiu-wu Bian

China

Antonio Bianchi

Italy

Maria Biazevic

Brazil

Hemant Kumar Bid

USA

Ettore Bidoli

Italy

Andreas Bikfalvi

France

Adrian Billeter

Germany

Julia Billiard

USA

Caroline Billingsley

USA

Helgi Birgisson

Sweden

Annamaria Biroccio

Italy

Oliver Blanck

Germany

Carmen Blanco-Aparicio

Spain

Gijs Bleijenberg

Netherlands

Elisabeth Bloemena

Netherlands

Josip Blonder

USA

Francesco Boccardo

Italy

Stefania Boccia

Italy

Michael Bodmer

Switzerland

Mogens Karsbøl Boisen

Denmark

Edwin Bölke

Germany

Catherine Bond

Australia

Mathieu Boniol

France

Shirin Bonni

Canada

Paul Bonnitcha

Australia

Maria Grazia Borrello

Italy

Magne Børset

Norway

Fred Bosman

Switzerland

Celine Bossard

France

Rémy Bosviel

France

Edoardo Botteri

Italy

Jan Bouchal

Czech Republic

Eric Bouffet

Canada

Jean-Christophe Bourdon

UK

Liam Bourke

UK

Roger Bourne

Australia

Hamouda Boussen

Tunisia

Anne-Marie Bouvier

France

Eric Bow

Canada

Kjetil Boye

Norway

Grace Bradley

Canada

Patrick Bradley

UK

Lucia Braga

Brazil

Walter Braga

Brazil

Elena Ioana Braicu

Germany

Johann Brandes

USA

Vasiliki Bravou

Greece

Rolf Brekken

USA

Darren Brenner

Canada

David Brindley

Canada

Cathrin Brisken

Switzerland

Robert Bristow

Canada

Alexander Brodsky

USA

Sabine Brookman-May

Germany

James Broome

USA

Linda Brown

USA

Brigid Browne

Australia

Kristine Browning

USA

Björn Brücher

Germany

Silke Brüderlein

Germany

Adam Brufsky

USA

Monika Brüggemann

Germany

Skjalg Bruheim

Norway

Ansgar Brüning

Germany

Hermann Brustmann

Austria

John Buatti

USA

Lukas Bubendorf

Switzerland

Frank Buchholz

Germany

Markus Buchler

Germany

Donald Buchsbaum

USA

Laurien Maria Buffart

Netherlands

Ida RK Bukholm

Norway

Ronald Bukowski

USA

Venkata Bulusu

UK

Nigel Bundred

UK

Joy Burchell

UK

Chris Burns

Australia

Howard Burris

USA

Jan Bussink

Netherlands

Benedetta Bussolati

Italy

Lesley Butler

USA

Alison Butt

Australia

Rita Byington

Brazil

Gabriella Cadoni

Italy

Solange Cadore

Brazil

Vincent Caggiano

USA

Guoxiang Cai

China

Mu-Yan Cai

China

Benjamin Cairns

UK

Viola Calabro

Italy

Daniele Calistri

Italy

Diego F Calvisi

Germany

Alfonso Calvo

Spain

Cesar Camara

Brazil

E Ramsay Camp

USA

Moray Campbell

USA

Giuseppina Campisi

Italy

Nicole Campos

USA

David Cano

Spain

Peter Canoll

USA

Robert Canter

USA

Gabriel Capella

Spain

Marzia Capelletti

USA

Ettore Capoluongo

Italy

Vera Cappelletti

Italy

Gabriele Capurso

Italy

Stefano Caramuta

Sweden

Eugen Carasevici

Romania

Brian Carr

Italy

Julie Carrier

Canada

Andreas Carus

Denmark

Joana Carvalho

Portugal

Silvia Carvalho

Portugal

Patrizia Casalini

Italy

Giuliana Cassinelli

Italy

Chiara Castelli

Italy

Enrique Castellon

Chile

Gabriella Castoria

Italy

Stefania Catalano

Italy

Gieri Cathomas

Switzerland

Loredana Cavalli

Italy

Arthur Cederbaum

USA

Wim Ceelen

Belgium

Brian Center

USA

Karen Cerosaletti

USA

Andres Cervantes

Spain

Hyuk-Jin Cha

South Korea

Karl Chai

USA

Zaher Chakhachiro

Lebanon

Chandan Chakraborty

Canada

Suzanne Chambers

Australia

Albert Chan

Hong Kong

Alexandre Chan

Singapore

Anthony Chan

Hong Kong

Chia-Hsin Chan

USA

David Chan

Hong Kong

Doris Chan

UK

Jennifer Chan

USA

Michael Chan

USA

Urmila Chandran

USA

Hong Chang

Canada

Jeffrey Chang

Taiwan

Julia Chang

USA

Kai-Ping Chang

Taiwan

Long-Sheng Chang

USA

Victor Chang

USA

Xiao Chang

USA

Yongsheng Chang

China

Yu-sun Chang

Taiwan

Jenny Chang-Claude

Germany

Pithi Chanvorachote

Thailand

Bridget Charbonneau

USA

Barbara Charbotel

France

Ajai Chari

USA

Jessica Charlet

USA

Frederic Charron

Canada

Naz Chaudary

Canada

Kazuaki Chayama

Japan

Maggie Cheang

USA

Satish Cheepala

USA

Meenakshi Chellaiah

USA

Bill Chen

USA

Chang-Han Chen

Taiwan

Chi-Ju Chen

Taiwan

Ching-Shih Chen

USA

Fei Chen

USA

Haiquan Chen

China

Hua Yun Chen

USA

I Chieh Chen

Taiwan

Kuen-Feng Chen

Taiwan

Li-Tzong Chen

Taiwan

Longbang Chen

China

Miaofen Chen

Taiwan

Ronald Chen

USA

Wantao Chen

China

Xin Chen

USA

Yi-Xing Chen

China

Jason Chia-Hsien Chen

Taiwan

Ji-Yen Cheng

Taiwan

Kuang-Hung Cheng

Taiwan

Liang Cheng

USA

Yan Cheng

USA

Chun Hei Antonio Cheung

Taiwan

Eric Chevet

France

Victoria Chia

USA

Maria-Dolores Chiara

Spain

Raffaella Chiaramonte

Italy

Chih-Yen Chien

Taiwan

Lung-Chang Chien

USA

Yoshitomo Chihara

Japan

Hiroki Chikumi

Japan

Sreenivasulu Chintala

USA

Simion Chiosea

USA

Han-Mo Chiu

Taiwan

Tai-Jan Chiu

Taiwan

Rowan Chlebowski

USA

Young-Rae Cho

USA

Alex Chou

USA

Christos Chouaid

France

Edward Chow

Singapore

Balram Chowbay

Singapore

Ezharul Chowdhury

Malaysia

Hans Christiansen

Germany

Thoralf Christoffersen

Norway

Hui-Ching Chuang

Taiwan

Shu-Chun Chuang

Taiwan

Eugene Chung

USA

Younh-Hwa Chung

South Korea

Gennaro Ciliberto

Italy

Graziella Cimino-Reale

Italy

Frank Claessens

Belgium

Susan Clare

USA

Megan Clarke

USA

Ramon Cleries

Spain

Anne-Marie Cleton-Jansen

Netherlands

Gary Clifford

France

Camille Cluze

France

Pierluigi Cocco

Italy

Kim Cocks

UK

Francesco Cognetti

Italy

Gennaro Colella

Italy

William Coleman

USA

Pedro A. Colinas

Argentina

Manuel Collado

Spain

Gisele Colleoni

Brazil

Laura Collins

USA

Yu-Sheng Cong

China

Stephen Connolly

UK

Patricia Coogan

Uzbekistan

Deirdre Coombe

Australia

Brenda Coomber

Canada

Kathleen Cooney

USA

Timothy Cooper

USA

Wendy Cooper

UK

Suzanne Coopey

USA

Giacomo Corrado

Italy

Ricardo Correa

USA

Renzo Corvo

Italy

Sarah Coupland

UK

Sébastien Couraud

France

Rita Cowell

USA

David Cox

France

M Christina Cox

Italy

Johannes Coy

Germany

Renata Cozzi

Italy

Holger Cramer

Germany

Ian Cree

UK

Anne Cress

USA

Rosemary Cress

USA

Jo Cresswell

UK

Jennifer Croke

Canada

Colleen Croniger

USA

Nicholas Cross

UK

Sidney Croul

Canada

David Crowe

USA

Vincent Cryns

USA

Brandi Cuevas

USA

Zoran Culig

Austria

Brian Cummings

USA

Douglas Curran-Everett

USA

Rakefet Czerninski

Israel

Adriana Da Silva-Conforti

Brazil

David Dabbs

USA

Gabi Dachs

New Zealand

Toos Daemen

Netherlands

Kiran Dahiya

India

Guang-Hai Dai

China

Qi Dai

USA

Yun Dai

USA

Maria Grazia Daidone

Italy

Maria Dalamaga

Greece

Sarah D'Alessandro

Italy

Jeanette Daly

USA

Megan Daly

USA

Irene Dambruoso

Italy

Nadia Dandachi

Austria

Peter Daniel

Germany

Marios Daskalopoulos

Greece

Adil Daud

USA

Sayeema Daudi

USA

Leonor David

Portugal

Jean-Luc Davignon

France

Ian Dayes

Canada

Roberta De Angelis

Italy

Michele De Bortoli

Italy

Giulia De Falco

Italy

Emma De Feo

Italy

Marieke de Graaff

Netherlands

Ivana de La Serna

USA

Manuel de Las Heras

Spain

Paulo de Sepulveda

France

Marco de Velasco

Japan

Gabriella De Vita

Italy

Chiara De Waure

Italy

Natasha Deane

USA

Bisrat Debeb

USA

Luc Defreyne

Belgium

Mario Del Rosso

Italy

Sinead Delany

South Africa

Alain Demers

Canada

Meltem Demirel Kars

Turkey

Carsten Denkert

Germany

Elizabeth Dennett

New Zealand

Melissa DeRycke

USA

Christèle Desbois-Mouthon

France

Anjali Deshpande

USA

Dominic Desiderio

USA

Alexander Deutsch

Austria

Beena Devi

Malaysia

Jennifer Devlin

Australia

Arun Dharmarajan

Australia

Giovan Giuseppe Di Costanzo

Italy

Marianna Di Martino

Italy

Elena Diaz

USA

Goberdhan Dimri

USA

Yu Ding

USA

Marina Diniz

Brazil

Andreas Dinkel

Germany

Luc Dirix

Belgium

Carmen Desirée Dirksen

Netherlands

Sharon Diskin

USA

Amanda Dixon-McIver

New Zealand

Bahia Djerdjouri

Algeria

Katalin Dobra

Sweden

Pauline Dobson

UK

Daniel R Doerge

USA

Hillaire Dominique

France

Massimo Donadelli

Italy

Xuesen Dong

Canada

Jessica Donington

USA

Claire Louise Donohoe

Ireland

Shashikiran Donthamsetty

USA

Keith Dookeran

USA

Thilo Dork

Germany

Eleonora Dorsi

Brazil

Bill Dougall

USA

Ryan Dowling

Canada

Amy Downing

UK

Tommaso Dragani

Italy

Jennifer Drahos

USA

George Dranitsaris

Canada

Jean-Luc Dreyer

Switzerland

Xiang Du

China

Xianglin Du

USA

Anna Dubrovska

Germany

François Ducray

France

Volker Duda

Germany

Alfonso Duenas-Gonzalez

Mexico

Hugues Duffau

France

David Duffy

Australia

Jonathan Duke-Cohan

USA

Jehan Dupuis

France

Polat Dursun

Turkey

Lars Dyrskjot

Denmark

Petras Dzeja

USA

Georgie Eapen

USA

Charles Eberhart

USA

Jan Eberth

USA

Sam Egger

Australia

Ann Marie Egloff

USA

David Eisenstat

Canada

Wolfgang Eisterer

Austria

Paul Ekert

Australia

Donatus Ekwueme

USA

Mohammed El Mzibri

Morocco

Osman El-Maarri

Germany

Lynne Elmore

USA

Marwan El-Sabban

Lebanon

Adam Elzagheid

Libya

Ehab Elzayat

Canada

Makoto Emoto

Japan

Diana English

USA

Meira Epplein

USA

Marie-Louise Essink-Bot

Netherlands

Arash Etemadi

USA

Leina Etto

Brazil

Matthias Evert

Germany

Francesco Fabbri

Italy

Muller Fabbri

USA

Antonio Facchiano

Italy

Oluwole Fadare

USA

Joanna Fair

USA

Diane Fairclough

USA

Ilaria Falciatori

USA

Luca Faloppi

Italy

Hong Fan

China

Jia Fan

China

Fu-Min Fang

Taiwan

Michael Fant

USA

Mark Faries

USA

Rae Farnsworth

Australia

Peter Fasching

Germany

Gilbert Faure

France

Gennaro Favia

Italy

Stefano Fedele

UK

Tanja Fehm

Germany

Harriet Feilotter

Canada

Andrew Feinberg

USA

Michael Feldman

USA

Claudio Feliciani

Italy

Antonio Feliciello

Italy

Emanuela Felley-Bosco

Switzerland

Nicola Fenderico

Italy

Annika Fendler

Germany

Feng Feng

USA

Ping Feng

China

Youji Feng

China

Cristiano Ferlini

USA

Narcis Fernandez-Fuentes

UK

Gabriella Ferrandina

Italy

Francesco Ferrara

Italy

Irene Ferrer

Spain

Robert Ferris

USA

Leah Ferrucci

USA

Tania Fiaschi

Italy

Gilberto Filaci

Italy

Ali Filiz

Turkey

Gaetano Finocchiaro

Italy

Wolfgang Fischbach

Germany

Matthias Fischer

Germany

Frank Fleischer

Germany

Ian Fleming

UK

Emmy Fleuren

Netherlands

Michael Flister

USA

Michael Foerster

Germany

Emmanouil Fokas

Germany

Marco Folini

Italy

Peter Fong

New Zealand

Caterina Fontanella

Germany

Andres Forero

USA

Jonathan Forsberg

USA

Kathleen Foster

USA

William Foulkes

Canada

Kathleen Fox

USA

Mario Fraga

Spain

Adam Enver Frampton

UK

Renato Franco

Italy

Philippe Frank

USA

Jeff Franklin

USA

Renty Franklin

USA

Alessandra Franzetti Pellanda

Switzerland

Antonio Frassoldati

Italy

Sarah Freemantle

USA

Pim French

Netherlands

Sara Frias

Mexico

Jeff Frost

USA

Fenghua Fu

China

Liwu Fu

China

Sau Nga Fu

Hungary

Siqing Fu

USA

Wei-Neng Fu

China

Susanne Fuessel

Germany

Barbara Fuhrman

USA

Tomoaki Fujioka

Japan

Naoya Fujita

Japan

David Fuks

France

Manabu Fukumoto

Japan

Peter Fuller

Australia

Junji Furuse

Japan

Mayuko Furuta

Japan

Masayuki Futagami

Japan

Anthony Fyles

Canada

Michael Gabriel

Austria

Gianluca Gaidano

Italy

Timo Gaiser

Germany

Stefanie Galban

USA

Margherita Gallicchio

Italy

Valerio Gallotta

Italy

Adelina Gama

Portugal

Angelo Gamez-Pozo

Spain

Marilie Gammon

USA

Paolo Gandellini

Italy

Apar Kishor Ganti

USA

Feng Gao

USA

Jian-Li Gao

China

Zhancheng Gao

China

Juan F Garcia

Spain

Angel Garcia Martin

Spain

Manuela Gariboldi

Italy

Scott Garrett

USA

Pedro Gascon

France

Anna Gasperi-Campani

Italy

Valter Gattei

Italy

Laura Gatti

Italy

Jean Gaudart

France

Luke Gaughan

UK

Maria Gazouli

Greece

Haiyan Ge

China

Donato Gemmati

Italy

Huimin Geng

USA

Petra Georg

Austria

Alex Georgakilas

Greece

Vassilis Georgoulias

Greece

Robert Getzenberg

USA

Ravit Geva

Israel

Farid Ghadessy

Singapore

Hazem Ghebeh

Saudi Arabia

Paola Ghiorzo

Italy

François Ghiringhelli

France

Constantinos Giaginis

Greece

Francesco Gianfagna

Italy

Elisa Giannoni

Italy

Spencer Gibson

Canada

Roben Gieling

UK

Gretchen Gierach

USA

Miguel Gil Gil

Spain

Sebastien Gilbert

Canada

Anna Gillio-Tos

Italy

Massimo Gion

Italy

Elisa Giovannetti

Netherlands

Catia Giovannini

Italy

Mirella Giovarelli

Italy

Olivier Gires

Germany

Leonard Girnita

Sweden

David Gius

USA

Michael Gnant

Austria

James Goedert

USA

Vikas Goel

USA

Asieh Golozar

USA

Isabel Gomez Monterrey

Italy

Vinai Gondi

USA

Aihua Gong

China

Zhihong Gong

USA

Lou Gonsalves

USA

Ellen Goode

USA

Pierre Goovaerts

USA

Srinivas Gopala

India

Ivan Gorlov

USA

Bohdan Gorski

Poland

Paul Goss

USA

Martin Götte

Germany

Corinne Gower-Rousseau

France

Norbert Graf

Germany

Begona Grana

Spain

Dan Granberg

Sweden

Michal Grat

Poland

William Greenhalf

UK

Thomas Griffith

USA

Giovanni Grignani

Italy

Bogdan Grigoriu

Romania

Catherine Grillon

France

Daniela Grimm

Denmark

Peter Grimminger

USA

Rachel Grisham

USA

Patrick Grohar

USA

Tove Gronroos

Finland

Nelleke Gruis

Netherlands

Eva Grunfeld

Canada

Viktor Grünwald

Germany

Sofia Gruvberger Saal

Sweden

Jian Gu

USA

Jianxin Gu

China

Xiaoxiang Guan

China

Xin-Yuan Guan

Hong Kong

Mickael Guedj

France

Vince Guerriero

USA

Massimo Guidoboni

Italy

Evin Gulbahce

USA

Ulrich Güller

Switzerland

Cheng Guo

China

Haiyan Guo

China

Ping Guo

USA

Yajun Guo

China

Vineet Gupta

USA

Ali Gure

Turkey

Simona Gurzu

Romania

Arthur Gutierrez-Hartmann

USA

Daniel Hackett

Australia

Christina Hackl

Germany

Rami Haddad

USA

Sassan Hafizi

UK

Jeffrey Hager

USA

Jana Halamkova

Czech Republic

Christa Haldrup

Denmark

Janet Hall

France

Nathan Hall

USA

Peter Hall

UK

Sigrun Hallmeyer

USA

Stephen Halloran

UK

Balazs Halmos

USA

Dolores Hambardzumyan

USA

Ghassan Hamra

France

Hye-Jung Han

USA

Junqing Han

China

Mingyong Han

China

Xiaofeng Han

USA

Ze-Guang Han

China

Sanshiro Hanada

Japan

David Hancock

UK

Nicholas Hand

USA

Claus Hanusch

Germany

Azizul Haque

USA

W Elaine Hardman

USA

Chuck Harrell

USA

Jenine Harris

USA

Hannah Harrison

UK

Tiffiney Hartman

USA

Susanne Hartwig

France

Hideki Hashimoto

Japan

Ursula Hasler-Strub

Switzerland

Mohamed Hassan

Germany

Georgia Hatzivassiliou

USA

John Hays

USA

Aiwu He

USA

Dongmei He

China

Liru He

China

Lionel Hebbard

Australia

Shantel Hébert-Magee

USA

Suzanne Hector

Ireland

Max Heiland

Germany

Florian Heitz

Germany

Ellen Heitzer

Austria

Karl Erik Hellstrom

USA

Rui Henrique

Portugal

Lynn Henry

USA

John Henson

USA

Jesus Hernandez

Spain

Patries Herst

New Zealand

Wolfgang Heuwieser

Germany

Tina Hieken

USA

Arnold Hill

Ireland

Georgios Hillas

Greece

Sally Hinchliffe

UK

Sebastian Hinde

UK

Victoria Hinder

New Zealand

Kei Hirai

Japan

Asahi Hishida

Japan

Miguel Hissa

Brazil

Han Kiat Ho

Singapore

Jessica Hoell

Germany

Camilla Hoff

Denmark

Kate Hoffman

USA

Leo Hofland

Netherlands

Janneke Hogervorst

Netherlands

Veronica Höiom

Sweden

Gregory Holt

USA

Masao Honda

Japan

Friedemann Honecker

Germany

Chi-Chen Hong

USA

Wanjin Hong

Singapore

Michael Höpfner

Germany

Craig Horbinski

USA

Amanda Hordern

Australia

Hidehito Horinouchi

Japan

Willi Horner-Johnson

USA

Dieter Hörsch

Germany

Terzah Horton

USA

Peter Hosein

USA

Isamu Hoshino

Japan

Jiazhou Hou

China

Ming-Feng Hou

Taiwan

Ming-Hong Hou

China

Roch Houot

France

Carrie House

USA

Harrison Howard

USA

William Hrushesky

USA

Cheng-Lung Hsu

Taiwan

Cheng-Ming Hsu

Taiwan

Chaosu Hu

China

Yanzhong Hu

China

Annie Huang

Canada

Bin Huang

USA

Chao-Cheng Huang

Taiwan

Hsuan-Ying Huang

Taiwan

Kuo-How Huang

Taiwan

Miriam Huang

USA

Peng Huang

USA

Rwei-Fen Huang

Taiwan

Wenlin Huang

China

Xiaoyi Huang

USA

Xinsheng Huang

China

Laurie Hudson

USA

Kay Huebner

USA

Judith Hugh

Canada

Lijian Hui

China

Michael Hummel

Germany

Ivan Hung

Hong Kong

Dezheng Huo

USA

Fazlul Huq

Australia

Chung Feng Hwang

Taiwan

Per Hydbring

USA

Carolina Ianuale

Italy

Merdol Ibrahim

UK

Naoki Ikenaga

USA

Martin Illemann

Denmark

Zsolt Illes

Hungary

Evgeny Imyanitov

Russian Federation

Stefano Indraccolo

Italy

Hidenori Inohara

Japan

Manami Inoue

Japan

Paul Insel

USA

Arvids Irmejs

Latvia

Mohamad-Rodi Isa

Malaysia

Sofie Isebaert

Belgium

Haku Ishida

Japan

Shinji Ishikawa

Japan

Toshihisa Ishikawa

Japan

Mitsuru Ishizuka

Japan

Yuri Ito

Japan

Orit Itzhaki

Israel

Fabio Iwamoto

USA

Hirotaka Iwase

Japan

Tomoko Iwata

UK

Gopalakrishna Iyer

Singapore

Reza Izadpanah

USA

William Jacot

France

Anders Jakobsen

Denmark

Marko Jakopovic

Croatia

Venkatakrishna Jala

USA

Ijaz Jamall

USA

Margit Janát-Amsbury

USA

Ramunas Janavicius

Lithuania

Jin Sung Jang

USA

Joung Jang

South Korea

Andrea Janikova

Czech Republic

Judith Jans

Netherlands

Radoslaw Januchowski

Poland

Thierry Jarde

Australia

Delphine Javelaud

France

Jaroslav Jelinek

USA

Jiiang-Huei Jeng

Taiwan

Wen-Juei Jeng

Taiwan

Randy Jensen

USA

Dennis Jeppesen

Denmark

Manisha Jhamb

USA

Guixiang Ji

China

Hongbin Ji

China

Junfang Ji

USA

Yuan Ji

China

Chuanlu Jiang

China

Bilian Jin

USA

Chunlei Jin

USA

Victor Jin

USA

Daewoong Jo

USA

Newell Johnson

Australia

Nicholas Johnson

UK

Resa M Jones

USA

Bharat Joshi

USA

Deepti Joshi

India

Anthony Joshua

Canada

Manfred Jücker

Germany

Alain Jung

France

Young Jung

South Korea

Achim Jungbluth

USA

Humam Kadara

USA

Bernd Kaina

Germany

Hiroshi Kajiya

Japan

Megan Kalambo

USA

Vaijayanti Kale

India

Enikoe Kallay

Austria

Takao Kamai

Japan

Ashish Kamat

USA

Erdinc Kamer

Turkey

Takeharu Kanazawa

Japan

Eleanor Kane

UK

Yasuhiko Kaneko

Japan

Melissa Kang

USA

Rajani Kanteti

USA

Kenneth Kao

Canada

Anil Kapoor

Canada

Gauri Kapoor

India

Matthias Kappler

Germany

Andreas Karachristos

USA

Nikos Karamanos

Greece

Sara Karami

USA

Anjali Karande

India

Aly Karsan

Canada

Efstathios Kastritis

Greece

Kota Katanoda

Japan

Venkat Katkoori

USA

Masaru Katoh

Japan

Ruth Katz

USA

Steven Katz

USA

Balveen Kaur

USA

Dara Kavanagh

Ireland

Patricia Keely

USA

Lital Keinan-Boker

Israel

Ameeta Kelekar

USA

Gisela Keller

Germany

Darshan Kelley

USA

Mark Kelley

USA

Lukas Kenner

Austria

Dermot Kenny

Ireland

Alex Kentsis

USA

Robert Kerbel

Canada

Stephanie Kermorgant

UK

Kayvan Keshari

USA

Youmna Kfoury

USA

Levon Khachigian

Australia

Mohamed Kharfan-Dabaja

USA

Abdel-Majid Khatib

France

Hung Khong

USA

Hippokratis Kiaris

Greece

Eiji Kikuchi

Japan

Dae-Ghon Kim

South Korea

Joseph Kim

USA

Kevin Kim

USA

Kyubo Kim

South Korea

Sangmi Kim

USA

Sang-Yong Kim

South Korea

Seung Up Kim

South Korea

Eric Kimchi

USA

Merel Kimman

Australia

Sheetal Kircher

USA

Tadaaki Kirita

Japan

Laurie Kirstein

USA

Kazushi Kishi

Japan

Yoji Kishi

Japan

Wolfram Klapper

Germany

Giseli Klassen

Brazil

Jean Klastersky

Belgium

Martin Klein

Netherlands

Miriam Kleiter

Austria

Jennifer Klemp

USA

Thomas Klonisch

Canada

Stian Knappskog

Norway

Beatrice Knudsen

USA

Sakari Knuutila

Finland

Ying-Chin Ko

Taiwan

Nozomu Kobayashi

Japan

Sebastian Kobold

Germany

Arend Koch

Germany

Roger Kockelbergh

UK

Viktor H Koelzer

Switzerland

Reinhard Kofler

Austria

Kimitoshi Kohno

Japan

Takashi Kohno

Japan

Norihiro Kokudo

Japan

Arunark Kolipaka

USA

Christian Kollmannsberger

Canada

Shunsuke Kondo

Japan

Dejuan Kong

USA

Konstantinos Konstantopoulos

USA

József Kónya

Hungary

Ja Seung Koo

South Korea

Liudmila Korkina

Italy

Christos Kosmas

Greece

Madhuri Koti

Canada

Athanasios Kotsakis

Greece

Zaklina Kovacevic

Australia

Richard Koya

USA

Shingo Kozono

Japan

Mori Krantz

USA

Mechthild Krause

Germany

Andreas Krieg

Germany

Lasse Sommer Kristensen

Denmark

Nils Kroeger

Germany

Judith Kroep

Netherlands

Feride Kroepil

Germany

Jennifer Kryworuchko

Canada

Lisha Kuang

USA

Greg Kubicek

USA

Mikael Kubista

Sweden

Omer Kucuk

USA

Donald Kufe

USA

Maria Kukuruzinska

USA

Shalini Kulasingam

USA

Dagmar Kulms

Germany

Ronald Kumon

USA

Shekhar Kumta

Hong Kong

Gopal Kundu

India

Chikara Kunisaki

Japan

Han-Pin Kuo

Taiwan

Peter Kuppen

Netherlands

Moni Abraham Kuriakose

USA

Hiroshi Kurita

Japan

Kazuhiko Kurozumi

Japan

Hironori Kusano

Japan

Boris Kuvshinoff

USA

Fabrice Kwiatkowski

France

Robert Kypta

UK

George Kyrgias

Greece

Athanassios Kyritsis

Greece

Marie-Christine Kyrtsonis

Greece

Mario Lacouture

USA

Sara Ladu

Italy

Robert Lafrenie

Canada

Chann Lagadec

France

Hung-Cheng Lai

Taiwan

Ite Laird-Offringa

USA

Vaia Lambadiari

Greece

Diether Lambrechts

Belgium

Pavlos Lampropoulos

Greece

Sven Lang

Germany

Rupert Langer

Switzerland

Anton Langerak

Netherlands

Scott Langevin

USA

Cord Langner

Austria

Jacques Lapointe

Canada

Berta Laquente

Spain

Zvi Laron

Israel

William Laskin

USA

André Lavorato-Rocha

Brazil

Matthew Lawless

Ireland

Sigurd Lax

Austria

Robert Layfield

UK

Alexander Lazar

USA

Philipp Lechler

Germany

Daniele Lecis

Italy

Gilho Lee

Canada

Ho Yun Lee

South Korea

Hsin-Chen Lee

Taiwan

Ming-Fen Lee

Taiwan

Minyoung Lee

South Korea

Se-Hoon Lee

South Korea

Terry Lee

Canada

Brian Lehmann

USA

Nadja Lehwald

Germany

Marjut Leidenius

Finland

Katia Leite

Brazil

Marina Leite

Portugal

Dido Lenze

Germany

Marino Leon

USA

Leondios Leondiadis

Greece

Wojciech Leppert

Poland

Gregory Lesinski

USA

Laurent Lessard

USA

Eleonora Leucci

Denmark

David Levens

USA

Christopher Lewis

New Zealand

Jason Lewis

USA

Etienne Leygue

Canada

Hecheng Li

China

Jun Li

China

Li Li

China

Min Li

USA

Rui Li

China

Wenhua Li

China

Wenqing Li

China

Yaming Li

China

Yulin Li

Portugal

Zonghai Li

China

Zongjin Li

China

Lay Hoong Lian

Malaysia

Min Lian

USA

Houjie Liang

China

Jie Liang

USA

Evi Lianidou

Greece

Chaosheng Liao

Taiwan

John Liao

USA

Linda Liao

USA

Shu-Fen Liao

Taiwan

Massimo Libra

Italy

Alexandre Liccioni

Spain

Cornelia Liedtke

Germany

Tracy Lightfoot

UK

Wan-Teck Lim

Singapore

Chin-Yo Lin

USA

Jennifer Lin

USA

Lian-Jie Lin

China

Reigh-Yi Lin

USA

Zu-yau Lin

Taiwan

Volkhard Lindner

USA

Jun-Yang Liou

Taiwan

Lindsay Lipinski

USA

Wendy Lipworth

Australia

Bolin Liu

USA

Gentao Liu

China

Heli Liu

China

Houbao Liu

China

Huaitian Liu

USA

Jinyi Liu

China

Minmin Liu

USA

Shing-Hwa Liu

Taiwan

Tianshu Liu

China

Wei Liu

USA

Xian-kui Liu

China

Xuefeng Liu

USA

Yueyong Liu

USA

Yunpeng Liu

China

Ingrid Ljuslinder

Sweden

Adana Llanos

USA

Hui-Wen Lo

USA

Lorenzo Lo Muzio

Italy

Cristiana Lo Nigro

Italy

Adhemar Longatto Filho

Portugal

Lai Meng Looi

Malaysia

Alessia Lopergolo

Italy

Jose Luis Lopez Guerra

Spain

Jose Lorente

Spain

Ian Lorimer

Canada

Domenica Lorusso

Italy

Martin Loss

Germany

Wenhui Lou

China

Johanna Louhimo

USA

Stan Louie

USA

Fotios Loupakis

Italy

Aoife Lowery

Ireland

Chang-Hsien Lu

Taiwan

Shaolei Lu

USA

Xiaomei Lu

China

Yi Lu

China

Andreas Lubienski

Germany

Maria Lucibello

Italy

Alessandro Lugli

Switzerland

Arno Lukas

Austria

Eiliv Lund

Norway

Kenneth Lundstrom

Switzerland

Nuno Lunet

Portugal

Oleg Lunov

Germany

Wei Luo

China

Xunda Luo

USA

Eleonora Lusito

Italy

Craig Lynch

Australia

Heidi Lyng

Norway

Cynthia Ma

USA

ZhengLiang Ma

China

John MacLeod

Canada

Lavanya Madakasira

USA

Danilo Maddalo

USA

Taira Maekawa

Japan

Chihaya Maesawa

Japan

Derek Magee

UK

Rangaswamy Maggad

India

Dianna Magliano

Australia

Anthony Magliocco

USA

Fulvio Magni

Israel

Stefano Maria Magrini

Italy

Beatriz Maia

Brazil

Patrick Maier

Germany

Eugenio Maiorano

Italy

Michel Maira

Switzerland

Shishir Maithel

USA

Nik Makretsov

Canada

Katharina Malinowsky

Germany

David Malkin

Canada

Bibekanand Mallick

India

Joshua Mammen

USA

Kwan Man

China

Tarinee Manchana

Thailand

Julien Mancini

France

Manuela Mancini

Italy

Jeffrey Mann

Australia

Ferdinando Mannello

Italy

Jian-Hua Mao

USA

Claudiu Maragritescu

Romania

Virginie Marcel

UK

Caterina Marchiò

Italy

Michael Mark

Switzerland

Ben Markman

Australia

Athina Markou

Greece

Deborah Marsh

Australia

Angela Marten

Germany

John Martens

Netherlands

Guido Martignoni

Italy

Suzanne Martin

UK

Juan Martinez

Spain

Otoniel Martinez-Maza

USA

Miriam Martini

Italy

Jorge Martin-Perez

Spain

Gianluca Marucci

Italy

Daniela Marzioni

Italy

Eduardo Mascareno

USA

Valentina Masola

Italy

Stephen Master

USA

Nagahide Matsubara

Japan

Koichi Matsuda

Japan

Tasuku Matsuoka

Japan

David Mattson

USA

Milena Maria Maule

Italy

Loredana Mauro

Italy

Ross Maxwell

UK

Felicity EB May

UK

Arnulf Mayer

Germany

Eleni Mayson

Australia

Antonio Mazzocca

Italy

Guillermo Mazzolini

Argentina

Sandra McAllister

USA

David McCormick

USA

Michael McCulloch

USA

Jeannine McCune

USA

Olga McDaniel

USA

Laura McDonald

UK

Paul McDonald

Canada

Katherine McGlynn

USA

Liane McGlynn

UK

Bruce McKay

Canada

Mark McKeage

New Zealand

Sarah McLaughlin

USA

Alex McMahon

UK

Donald McMillan

UK

Lisa McShane

USA

Icro Meattini

Italy

Frederique Megnin-Chanet

France

Gill Mein

UK

Juan Manuel Mejia-Arangure

Mexico

Paula Meleady

Ireland

Antoine Melkane

USA

Rolf Mentlein

Germany

Domenico Franco Merlo

Italy

Ray Merrill

USA

Axel Merseburger

Germany

Stefania Meschini

Italy

Giulio Metro

Italy

Adam Metwalli

USA

Mark Meuth

UK

Stefan Meyer

UK

Paul Meyers

USA

Delia Mezzanzanica

Italy

Christine Miaskowski

USA

Francesca Micci

Norway

Gaetane Michaud

USA

Olivier Micheau

France

Pellegrino Michele

Italy

Gerald Mickisch

Germany

Antimo Migliaccio

Italy

Michele Davide Mignogna

Italy

Thomas Mikeska

Australia

Karin Milde-Langosch

Germany

Jeremy Miles

USA

Barbra Miller

USA

Giuseppe Milone

Italy

Gabriel Minarik

Slovakia

Miriam Mints

Sweden

Lisa Mirabello

USA

Clelia Miracco

Italy

Rajakishore Mishra

USA

Wilhelm Mistiaen

Belgium

Futakuchi Mitsuru

Japan

Yoshihiro Miyasaka

Japan

Isao Miyashiro

Japan

Kazuo Miyashita

Japan

Hiroshi Miyata

Japan

Yoshihiko Miyata

Japan

Toru Mizuguchi

Japan

Ali Mobasheri

UK

Holger Moch Moch

Switzerland

Helmout Modjtahedi

UK

Markus Moehler

Germany

Mohammadreza Mohebbi

Austria

Tudor Moldoveanu

USA

Alessio Molfino

Italy

Sonia Molina-Pinelo

Spain

Francesca Molinari

Switzerland

Miguel-Angel Molina-Vila

Spain

Ute Moll

USA

Chiara Mondello

Italy

Maria Monne

Italy

Clara Montagut

Spain

Ali Montazeri

Iran

Luis Montuenga

Spain

Ronald Moore

Canada

Roger Moorehead

Canada

Anthony Moorman

UK

Ann Moormann

USA

Vanessa Morais Freitas

Brazil

Violaine Moreau

France

Victor Moreno

Spain

Meredith Morgan

USA

Richard Morgan

UK

Mitsuru Mori

Japan

Chikao Morimoto

Japan

Ian Morison

New Zealand

Laura Moro

Italy

Michele Mortarino

Italy

Thomas Moss

USA

Susan Moug

UK

Aubin Moutal

France

Robert M Mroz

Poland

Kenneth Muir

UK

Ken-Ichi Mukaisho

Japan

Dhanya Mullassery

UK

Jean-Marc Muller

France

Arndt-Christian Müller

Germany

Arasambattu Kannan Munirajan

India

Sandra Muñoz-Galván

Spain

Jordi Muntané

Spain

Yoshiki Murakami

Japan

Satoru Murata

Japan

Satoshi Murata

Japan

Thomas E Mürdter

Germany

Gwen Murphy

USA

Graeme Murray

UK

Adele Murrell

UK

Maureen Murtaugh

USA

Muhammed Murtaza

Pakistan

Joshua Muscat

USA

Orlando Musso

France

Keun Na

South Korea

Ivan Nabi

Canada

Ramaiah Nagaraja

USA

Kazunori Nagasaka

Japan

Eva Nagele

Austria

Iris Nagtegaal

Netherlands

Hareth Nahi

Sweden

Clara Nahmias

France

Hiroshi Nakagawa

USA

Noboru Nakaigawa

Japan

Noriko Nakajima

Japan

Kohei Nakata

Japan

Yoshihiro Nakatani

USA

Naoki Nakaya

Denmark

Tomoki Nakaya

Japan

Meijin Nakayama

Japan

Tomio Nakayama

Japan

Kiyoshi Nakazaki

Japan

Joo-Hyun Nam

South Korea

Atsushi Nambu

Japan

Yoshio Naomoto

Japan

Marius Nap

Netherlands

Mayumi Naramura

USA

Nisha Narayan

Australia

Ajit Narayanan

New Zealand

Aziza Nassar

USA

Rachael Natrajan

UK

Ruta Navakauskiene

Lithuania

Roman Nawroth

Germany

Bojan Nedovic

Serbia

Eva Negri

Italy

Francesca Negri

Italy

David Nelson

USA

Mark Nelson

USA

John Nemunaitis

USA

Marika Nestor

Sweden

Heli Nevanlinna

Finland

Patrick Neven

Belgium

Bart Neyns

Belgium

Quan Sing Ng

Singapore

Simon Ng

Hong Kong

Nam Nguyen

USA

Ruby Nguyen

USA

Bing Ni

China

Craig Nichols

USA

Theo Nicolaides

USA

Kim Nicolaije

Netherlands

Jan Nicolay

Germany

Giorgia Nicolini

Switzerland

Ryan Niederkohr

USA

Hans Nijman

Netherlands

Hiroshi Nishida

Japan

Reiki Nishimura

Japan

Benjamin Nisman

Israel

Donato Nitti

Italy

Xiaohui Niu

China

Maximilian Niyazi

Germany

Vincent Njar

USA

Sisse Helle Njor

Denmark

Emeka Nkenke

Germany

Elfriede Noessner

Germany

Stig Nordling

Finland

Marianne Nordsmark

Denmark

Nicola Normanno

Italy

Francesco Novelli

Italy

Isao Nozaki

Japan

Fabio Nunes

Brazil

Ula Nur

UK

Heather Ochs-Balcom

USA

Kathleen O'Connor

USA

Norma O'Donovan

Ireland

Peter Oehr

Japan

Shuji Ogino

USA

Olorunseun Ogunwobi

USA

Ralf Ohlinger

Germany

Nitin Ohri

USA

Masayuki Ohtsuka

Japan

Takao Ohtsuka

Japan

Hirohisa Okabe

Japan

Hideho Okada

USA

Seiji Okada

Japan

Alicia Okines

UK

Tomohisa Okuma

Japan

Ake Oldberg

Sweden

Peter O'Leary

Ireland

Benigna Oliveira

Brazil

Cristina Oliveira

Brazil

Leticia Oliveira-Ferrer

Germany

Dorte Olsen

Denmark

Jordan Olson

USA

Kim O'Neill

USA

Evan Ong

USA

Hiroshi Onishi

Japan

Michael O'Rorke

UK

Nick Orr

UK

Gert Ossenkoppele

Netherlands

Marie Stampe Ostenfeld

Denmark

Eleanor O'Sullivan

Ireland

Megan Othus

USA

Sandra OToole

Australia

Penelope Ottewell

UK

Ouathek Ouerfelli

USA

Iwata Ozaki

Japan

Enver Ozer

Venezuela

Reetesh Pai

USA

Tuya Pal

USA

Fabrizio Palitti

Italy

Massimo Pancione

Italy

Siyaram Pandey

Canada

Hardev Pandha

UK

Giuseppe Pandini

Italy

Carolina Panis

Brazil

Michel Paques

France

Olivier Pardo

UK

Joana Paredes

Portugal

David Parham

USA

Purvish Parikh

India

Catherine Park

USA

Il-Seok Park

South Korea

In Hae Park

South Korea

Won Sang Park

South Korea

Maria Paola Paronetto

Italy

Luca Parrillo

Italy

Valeria Pascale

Italy

Boris Pasche

USA

Annette Paschen

Germany

Freda Passam

Australia

Antonio Luigi Pastore

Italy

Jaromir Pastorek

Slovakia

Roberta Pastorino

Italy

Jitesh Patel

USA

Kepal Patel

USA

Manish Patel

USA

Sujata Patil

USA

Nicholas Pavlidis

Greece

Peter Pedersen

USA

Petra Peeters

Netherlands

Jing Pei

China

Luc Pellerin

Switzerland

Claudio Pelucchi

Italy

Cristina Pena

Spain

Lirong Peng

USA

Lu Peng

China

Marzia Pennati

Italy

Andrea Peracino

Italy

Paola Perego

Italy

Marcos Perini

Brazil

Daniele Perna

UK

Rosario Perona

Spain

Alexander Pertsemlidis

USA

Ulf Peters

USA

Kevin Petrecca

Canada

Fausto Petrelli

Italy

Marco Petrillo

Italy

Karl-Ulrich Petry

Germany

Stefano Petti

Italy

W Jeffrey Petty

USA

Francesca Pica

Italy

Pier Paolo Piccaluga

Italy

Martin Pichler

Austria

Ada Piepoli

Italy

Jean-Yves Pierga

France

Marco Alessandro Pierotti

Italy

Guillaume Piessen

France

Kristian Pietras

Sweden

Albrecht Piiper

Germany

Robert Pilarski

USA

Dietmar Pils

Austria

Céline Pinheiro

Brazil

Hugo Pinheiro

Portugal

Paulo Pinheiro

USA

Chelsea Pinnix

USA

Giovanny Pinto

Brazil

Luis Pinto

Brazil

Kirstin Pirie

UK

Ana Planavila

Spain

George Plataniotis

UK

Robert Platt

Canada

Ruben Plentz

Germany

Francesco Plotti

Italy

John Plukker

Netherlands

Janice Pluth

USA

Osvaldo Podhajcer

Argentina

Juan Poggio

USA

Karen Pollok

USA

Vladimir Ponomarev

USA

Mirco Ponzoni

Italy

Maria Grazia Porpora

Italy

David Porter

New Zealand

Anna Potapova

Germany

Nancy Potischman

USA

Ryan Potts

USA

Mohamad Amin Pourhoseingholi

Iran

Samantha Pozzi

Italy

Aleix Prat

Spain

Guy Pratt

UK

Timothy Price

Australia

Anne Prieto

USA

Kathleen Pritchard

Canada

Valentina Profumo

Italy

Jenifer Prosperi

USA

Mariano Provencio

Spain

Sandi Pruitt

USA

Martin Pruschy

Switzerland

Hong Pu

USA

Gerald Pühse

Germany

Jean Louis Pujol

France

Ashok Pullikuth

USA

Hongryull Pyo

South Korea

Liao Qiande

China

Guilin Qiao

USA

Lun-Xiu Qin

China

Jiliang Qiu

China

Xiujuan Qu

China

Martin Quinn

UK

Alfredo Quinones-Hinojosa

USA

Elisabeth Quoix

France

Manuela Rabaglio

Switzerland

Milan Radovich

USA

Sinisa Radulovic

Serbia

Sajjad Rafiq

UK

Prabhakar Rajan

UK

Rajiv Rajani

USA

Moganty Rajeswari

India

Michal Rajski

Switzerland

Janusz Rak

Canada

Giuliano Ramadori

Germany

Steven Raman

USA

V Krishnan Ramanujan

USA

Pranela Rameshwar

USA

Kenneth Ramos

USA

Maria Ramos

Spain

Sophia Ran

USA

Leticia Rangel

Brazil

Nancy Ratner

USA

Tilman Rau

Germany

Azad Razack

UK

Francisco Real

Spain

Francisco X Real

Spain

Luis Miguel Real

Spain

Robert Rebhun

USA

Matejka Rebolj

Denmark

Shyam Reddy

USA

Thipparthi Reddy

USA

Anne Reeler

Denmark

Jennifer Reese

USA

Christian Regenbrecht

Germany

Mauricio Reginato

USA

Noemi Reguart

Spain

Jordi Remon Masip

Spain

Huan Ren

China

Oliver Renner

Spain

Miriam Reuschenbach

Germany

Françoise Révillion

France

Nicolas Reymond

France

Andy Reynolds

UK

Jose Riancho

Spain

Ana Sofia Ribeiro

Portugal

Dario Ribero

Italy

Jean-Ehrland Ricci

France

Micheler Richardson

USA

Luisel Ricks-Santi

USA

Brian Rini

USA

Gail Risbridger

Australia

Nesrine Rizk

Lebanon

Wasia Rizwani

USA

Trude Eid Robsahm

Norway

Sonia Rocha

UK

Benjamin Roche

France

Christoph Röcken

Germany

Andrew Roddam

UK

Franz Rödel

Germany

Richard Roden

USA

Monica Rodolfo

Italy

Rene Rodriguez

Spain

Claire Rodriguez-Lafrasse

France

Matthias Roessle

Switzerland

Sabine Rohrmann

Switzerland

Antti Roine

Finland

Federico Roncaroli

UK

Luca Roncucci

Italy

Ilse Rooman

Australia

Antonio Rosato

Italy

Rafael Rosell

Spain

Robert Roskoski

USA

Charles Rosser

USA

Matteo Rota

Italy

Arnaud Roth

Switzerland

Gael Roue

Spain

Kasper Rouschop

Netherlands

Caroline Rousseau

France

G Enrico Rovati

Italy

Luca Roz

Italy

Eric Rozet

Belgium

Lin Ruan

China

Qiang Ruan

China

Domenico Rubello

Italy

Volker Rudat

Saudi Arabia

Charles Rudin

USA

Paul Rudnick

USA

Maximilian Ruge

Germany

Antonio Russo

Italy

Paul Russo

USA

Mark Rutherford

UK

Gerard Rutteman

Netherlands

Eduard Ryschich

Germany

Min-Hee Ryu

South Korea

Lao Saal

Sweden

Seppo Saarelainen

Finland

Tiina Saarto

Finland

Hisataka Sabe

Japan

Mariusz Sacharczuk

Poland

Toshiaki Saeki

Japan

Manujendra Saha

Canada

Neeru Saini

India

Kazutaka Saito

Japan

Masao Saitoh

Japan

Yasunaru Sakuma

Japan

Toshiharu Sakurai

Japan

Daniel Salamon

Sweden

Roberto Salgado

Belgium

Athanasios Salifoglou

Greece

Natalie Sampson

Austria

Miguel Sanchez

USA

Rupninder Sandhu

USA

Juan Santibanez

Serbia

Mario Santinami

Italy

Sankar Sanyal

India

Anida Sarajlic

UK

Jann Sarkaria

USA

David Sarrio

UK

Aaron Sarver

USA

Hidefumi Sasaki

Japan

Norihiro Sato

Japan

Taroh Satoh

Japan

Sharon Savage

USA

Sunita Saxena

India

Susanna Scarpa

Italy

Mario Scartozzi

Italy

Reinhold Schäfer

Germany

Karl Schaller

Switzerland

Philip Schaner

USA

Tom Scharschmidt

USA

Dorthe Schaue

USA

Vera Schellerer

Germany

Arnaud Scherpereel

France

Gaia Schiavon

UK

Aaron David Schimmer

Canada

Anna Melissa Schlitter

Germany

Michael Schmid

UK

Carl Schmidt

USA

Marjanka Schmidt

Netherlands

Martina Schmidt

Netherlands

Fernando Schmitt

Portugal

Manfred Schmitt

Germany

Marc Schmitz

Germany

Hans-Joachim Schmoll

Germany

Abraham Schneider

USA

Helmut Schoellnast

Austria

Patricia Schoenlein

USA

Nathalie Scholler

USA

Mario Schootman

USA

Nina Schor

USA

Scott Schuetze

USA

Hildegard Schuller

USA

Beate Schultheis

Germany

Wolfgang Schulz

Germany

Udo Schumacher

Germany

Eric Schuur

USA

Heidi Schwarzenbach

Germany

Christian Schwentner

Germany

Marion Scoggins

USA

Andreas Scorilas

Greece

Carolyn Scott

Australia

Kieran Scott

Australia

Rodney Scott

Australia

Anna Ivana Scovassi

Italy

Siegfried Segaert

Belgium

Brahm Segal

USA

Eva Segelov

Australia

Bruno Ségui

France

Morgan Sellers

USA

Andrei Seluanov

USA

Gregg Semenza

USA

Boris Sepesi

USA

Simona Serini

Italy

Alexandre Serra

Germany

Gregor Sersa

Slovenia

Mukund Seshadri

USA

Cristiana Sessa

Switzerland

Yasuyuki Seto

Japan

Patricia Severino

Brazil

Tesa Severson

Netherlands

Alex Sgambato

Italy

George Sgourakis

Greece

Lorraine Shack

Canada

Chirayu Shah

USA

Shavali Shaik

USA

Masood Shammas

USA

Bin Shan

USA

Chidananda Sharma

India

Lena Sharp

Sweden

Richard Shaw

UK

Patricia Sheean

USA

Orla Sheils

Ireland

Malathy Shekhar

USA

Lin Shen

China

Trevor Shepherd

Canada

Karen Sherman

USA

David Sherr

USA

Sujit Sheth

USA

Kirti Shetty

Vanuatu

Lalita Shevde-Samant

USA

Runhua Shi

USA

Yongquan Shi

China

Paul Shiels

UK

Jin-Yuan Shih

Taiwan

Hideaki Shimada

Japan

Tomoyuki Shimada

Japan

Takashi Shimokawa

Japan

Narayan Shivapurkar

USA

Chang Shu

China

Brian Shuch

USA

Arti Shukla

USA

Suneet Shukla

USA

Zhang Shumin

China

Kenneth Siddle

UK

Heinz Sill

Austria

Paulo Silveira

Brazil

Tatjana Simic

Serbia

Alison Sinclair

UK

Amar Singh

USA

Pankaj Singh

USA

Rakesh Singh

USA

Daniel Sinnett

Canada

Ferenc Sipos

Hungary

Jennifer Sipos

USA

Fabrice Sircoulomb

Canada

Bhawna Sirohi

India

Tristan Sissung

USA

Dongrong Situ

China

Jens Siveke

Germany

Rod Skinner

UK

Ondrej Slaby

Czech Republic

Terry Slevin

Australia

Brian Slomovitz

USA

Artur Slupianek

USA

William Small Jr

USA

Peter Sminia

Netherlands

Jeffrey Smith

USA

Matija Snuderl

USA

Manuel Sobrinho Simões

Portugal

Tomotaka Sobue

Japan

Ole Solheim

Norway

Pamela Soliman

USA

Szilvia Solyom

USA

Sivagurunathan Somasundaram

USA

Fengju Song

China

Guisheng Song

USA

Yong Song

China

Pierre Sonveaux

Belgium

Prasanna Sooriakumaran

UK

Robert Sorge

USA

John Souglakos

Greece

Paul Span

Netherlands

Rachel Sparks

USA

Marijn Speeckaert

Belgium

Douglas Spitz

USA

Jeremy Squire

Canada

Kshitij Srivastava

USA

Rakesh Srivastava

USA

Dawn Stacey

Canada

Elvira Stacher

Austria

Sylvia Stadlmann

Switzerland

Jane Starczynski

UK

Giorgio Stassi

Italy

Georgios Stathopoulos

Greece

Jason Steel

USA

Diana Steinmann

Germany

Sally Stephenson

Australia

William Sterlacci

Austria

Anne Stey

USA

Charles Stiller

UK

Lucia Anna Stivala

Italy

Gabriele Stocco

Italy

Nikolas Hendrik Stoecklein

Germany

Robert Stoehr

Germany

Oliver Stoeltzing

Germany

Oliver Stoetzer

Germany

Guy Storme

Belgium

Alexander Stratigos

Greece

Deborah Stroka

Switzerland

Sofie Struyf

Belgium

Darrin Stuart

USA

Alina Sturdza

Austria

Michael Stürzl

Germany

Jyan-Gwo Su

Taiwan

Li-Jen Su

Taiwan

Rongjian Su

China

Kazushi Sugimoto

Japan

Haruhiko Sugimura

Japan

Kiminori Sugino

Japan

Iyad Sultan

Jordan

Asma Sultana

UK

Dan Sun

China

Shi-Yong Sun

USA

Malin Sund

Sweden

Karin Sundfeldt

Sweden

Hedwig Sutterluety-Fall

Austria

Akihiko Suzuki

Japan

Hiromu Suzuki

Japan

Michitaka Suzuki

Japan

Inge Marie Svane

Denmark

Irene Szijan

Argentina

Richard Szydlo

UK

Elda Tagliabue

Italy

Pierosandro Tagliaferri

Italy

Hsin-Hsiung Tai

USA

Katayama Takafumi

Japan

Chiaki Takahashi

Japan

Koji Takahashi

Japan

Shunji Takahashi

Japan

Masaaki Takamura

Japan

Yasuo Takano

Japan

Naoko Takebe

USA

Atsushi Takenaka

Japan

Mitsuhiro Takenoyama

Japan

Akinobu Taketomi

Japan

Hiroya Takeuchi

Japan

Nagio Takigawa

Japan

Yuichi Takiguchi

Japan

Maroulio Talieri

Greece

Anne Talvensaari-Mattila

Finland

Filippo Tamanini

Netherlands

Jun-Ichi Tamaru

Japan

Masahiko Tameda

Japan

Hani Tamim

Lebanon

Ernst Tamm

Germany

Daniel Tan

Singapore

Eng Huat Tan

Singapore

Lijie Tan

China

Motoyoshi Tanaka

Japan

Keiji Tanese

Japan

Chih-Hsin Tang

Taiwan

Hao Tang

China

Hua Tang

China

Jie Tang

China

Naping Tang

China

Ni Tang

China

Ping Tang

USA

Wei Tang

USA

Yaxiong Tang

China

Robert Tanguay

Canada

Satoshi Tanida

Japan

Tamara Tanos

Italy

Hou-Quan Tao

China

Junyan Tao

USA

Nadya Tarasova

USA

Rosemary Tate

UK

Ryosuke Tateishi

Japan

Helge Taubert

Germany

Niall Tebbutt

Australia

Beverly Teicher

USA

Julio Teixeira

Brazil

Gennadiy Telegeev

Ukraine

Achim Temme

Germany

Melissa Teo

Singapore

Alexei Tepikin

UK

Shuji Terai

Japan

Koji Teramoto

Japan

Luigi Terracciano

Switzerland

Carlo Terrone

Italy

Ugo Testa

Italy

Archana Thakur

USA

George Thalmann

Switzerland

Anne Thiebaut

France

Laure Thomas

France

Kyle Thompson

USA

Inger Thune

Norway

Jeffrey Tice

USA

Dirk Timmerman

Belgium

Ingeborg Tinhofer

Germany

Dina Tiniakos

Greece

Glen Titmarsh

UK

Pankaj Tiwari

USA

Yuji Toiyama

Japan

Amanda Toland

USA

Antonella Tomassetti

Italy

Anna Tomezzoli

Italy

Masaki Tomita

Japan

Minoru Tomizawa

Japan

Gail Tomlinson

USA

Joanna Tong

Hong Kong

Patricia Tonin

Canada

Matthew Topham

USA

Erkan Topkan

Turkey

Chiara Tordonato

Italy

Toshihiko Torigoe

Japan

Maria Lina Tornesello

Italy

Luigi Tornillo

Switzerland

Alicia Torriglia

France

Tibor Tot

Sweden

József Tóvári

Hungary

Eugene Toy

USA

Hidenori Toyoda

Japan

Anna Trauzold

Germany

Marco Tripodi

Italy

Giancarlo Troncone

Italy

Luan Truong

USA

George Sai-Wah Tsao

Hong Kong

Nicolas Tsavaris

Greece

Darjus Tschaharganeh

USA

Chin-Hsiao Tseng

Taiwan

Vassiliki Liana Tsikitis

USA

George Tsirakis

Greece

Koji Tsuboi

Japan

Katsuya Tsuchihara

Japan

Tung Yu Tsui

Germany

Takemasa Tsuji

USA

Jeffrey Tuan

Italy

Marco Tucci

Italy

Mutahir Tunio

Saudi Arabia

Ali Turhan

France

Nancy Turner

USA

Carl Tyler

USA

Jonathan Tyrer

UK

James Tysome

UK

Peter Udelnow

Germany

Yutaka Ueda

Japan

Kenichiro Uemura

Japan

Masaki Ueno

Japan

Zoe Uhry

France

Osamu Ukimura

USA

Shahid Umar

USA

Muhammad Umer

Pakistan

Roger Uren

Australia

Peralta Uroz

Spain

Giske Ursin

Norway

Josie Ursini-Siegel

Canada

Toshikazu Ushijima

Japan

Akihiko Usui

UK

Markku Vaarala

Finland

Maria Vaccaro

Argentina

Ratna Vadlamudi

USA

Manuel Valladares-Ayerbes

Spain

Sonia Vallet

Germany

François Vallette

France

Adriaan van den Brule

Netherlands

Peter van der Geer

USA

Kurt van der Speeten

Belgium

Paul van Diest

Netherlands

Yvette van Gestel

Netherlands

Toon van Gorp

Netherlands

Elke van Hoof

Belgium

Johan van Krieken

Netherlands

Jos van Pelt

Belgium

Catherine van Poznak

USA

Jean-Marc Vanacker

France

Barbara Vanderhyden

Canada

Andreas Varkaris

USA

Yogesh Vashist

Germany

Erik Vassella

Switzerland

Lars Vatten

Norway

Juergen Veeck

Germany

Marco Velasco-Velazquez

Mexico

Sriram Venneti

USA

Mukesh Verma

USA

Yvonne Versleijen-Jonkers

Netherlands

Therese Risom Vestergaard

Denmark

Rajeev Vibhakar

USA

Gregory Videtic

USA

Raffaella Villa

Italy

Paolo Villari

Italy

Elizabeth Vincan

Australia

Fabrizio Vincenzi

Italy

Francisco Vizoso

Spain

Erina Vlashi

USA

K David Voduc

Canada

Christina Voelkel-Johnson

USA

Arndt Vogel

Germany

Henrik von Euler

Sweden

Margaret von Mehren

USA

Jan Willem Voncken

Netherlands

Dirk Vordermark

Germany

Gerassimos Voutsinas

Greece

Semir Vranic

Bosnia and Herzegovina

Sven Wach

Germany

Heather M Wallace

UK

Timothy Wallace

USA

Jo Waller

UK

Thorsten Walles

Germany

Sherrie Wallington

USA

Binliang Wang

China

Chen Wang

USA

Cheng-Ping Wang

Taiwan

Daowen Wang

China

Dong Wang

China

Hsin-Ell Wang

Taiwan

Hua-Qin Wang

China

Jaw-Yuan Wang

Taiwan

Jean Wang

USA

Liang Wang

USA

Liang-Shun Wang

Taiwan

Mingwei Wang

China

Rong Fu Wang

China

Sophia Wang

USA

Timothy Wang

USA

Ting Wang

China

Yigang Wang

China

Yihong Wang

USA

Yuzhuo Wang

Canada

Zhao-Xia Wang

China

Zhou Wang

USA

John Ward

USA

Christopher Warlick

USA

Saman Warnakulasuriya

UK

Christina Warnecke

Germany

William Watson

Ireland

Kaaren Watts

Australia

Gabriel Wcislo

Poland

Scott Weed

USA

Guangwei Wei

China

Jun Wei

USA

Qiou Wei

USA

Kurt Weiss

USA

Alessandro Weisz

Italy

Scott Welford

USA

Ulrich Friedrich Wellner

Germany

Wanqing Wen

USA

Weihong Wen

China

Tamra Werbowetski-Ogilvie

Canada

Jelle Wesseling

Netherlands

Arne Westgaard

Norway

Cynthia Wetmore

USA

Hayley Whitaker

UK

Noel Whitaker

Australia

Eliza Whiteside

Australia

Theresa Whiteside

USA

Jeffrey Wickliffe

USA

Thomas Wight

USA

Chumpon Wilasrusmee

Thailand

Charlotte Wilhelm-Benartzi

UK

Neal Wilkinson

USA

Ann Williams

UK

Jennie Williams

USA

Matthew Williams

USA

Michelle Williams

USA

Anna-Lise Williamson

South Africa

Carlene Wilson

Australia

Joerg Wilting

Germany

Saskia Wilting

Netherlands

Boris Winterhoff

USA

Kerri Winters-Stone

USA

Joerg Wischhusen

Germany

Paul Wise

USA

Joachim Wiskemann

Germany

Philip Wong

Canada

Wai Wong

UK

Adisak Wongkajornsilp

Thailand

Craig Woodworth

USA

Curtis Wray

USA

Christina Wu

USA

Chung-Pu Wu

Taiwan

Chun-Te Wu

Taiwan

Erxi Wu

USA

Guo-Qing Wu

China

Hanping Wu

USA

Hsien-Ming Wu

Taiwan

Jinhui Wu

China

Lili Wu

China

Ming Wu

China

Xifeng Wu

USA

Youtong Wu

USA

Yu-Chung Wu

Taiwan

Bing Xia

China

Yunfei Xia

China

Feifei Xiao

USA

Helan Xiao

Canada

Hua Xiao

USA

Xiangwei Xiao

USA

Zhen Xiao

USA

Zhousheng Xiao

USA

Feng Xu

China

Guandong Xu

Australia

Jian Ming Xu

China

Jianmin Xu

China

Lin Xu

China

Ruiyun Xu

China

Yaji Xu

USA

Suayib Yalcin

Turkey

Toshiyuki Yamaji

Japan

Hidetaka Yamamoto

Japan

Hiroyuki Yamamoto

Japan

Masayuki Yamamoto

Japan

Taro Yamashita

Japan

Takashi Yamatodani

Japan

Takahiro Yamauchi

Japan

Hideya Yamazaki

Japan

Lunan Yan

China

Jin-Ming Yang

USA

Ling Yang

UK

Liu Yang

USA

Seung Ho Yang

South Korea

Tsung-Lin Yang

Taiwan

Xiang-Jiao Yang

Canada

Xiaojing Yang

USA

Yongping Yang

China

Yang Yao

China

Slav Yartsev

Canada

Kohichiroh Yasui

Japan

Kosei Yasumoto

Japan

Weimin Ye

Sweden

Herman Yeger

Canada

Chia Jui Yen

Taiwan

Rinat Yerushalmi

Israel

John Yim

USA

Takao Yokoe

Japan

Raymund Yong

USA

Seiichi Yoshimoto

Japan

Ramy Youssef

USA

Chao-Lan Yu

USA

Helena Yu

USA

Jau-Song Yu

Taiwan

Jian Yu

USA

Li-Rong Yu

USA

Nam Yu

USA

Onchee Yu

USA

Ri-Sheng Yu

China

Xianjun Yu

China

Xiuchun Yu

China

Xue Qin Yu

Australia

Geng Biao Yuan

China

Gelareh Zadeh

Canada

John Zalcberg

UK

Khalil Zaman

Switzerland

Rita Zamarchi

Italy

Gian Franco Zannoni

Italy

Robert Zeillinger

Austria

Yi-Xin Zeng

China

Zhao-Chong Zeng

China

Thorsten Zenz

Germany

Lixing Zhan

China

Yiqiang Zhan

USA

Baolin Zhang

USA

Guo-Jun Zhang

China

Jian Zhang

China

Jianjun Zhang

USA

Meixia Zhang

China

Mingfeng Zhang

USA

Paul Zhang

USA

Qingfu Zhang

China

Xu Dong Zhang

Australia

Zhen Zhang

USA

Hongling Zhao

USA

Kuai-le Zhao

China

Weili Zhao

China

Xiangshan Zhao

USA

Dexian Zheng

China

Hua-Chuan Zheng

China

Limin Zheng

China

Shusen Zheng

China

Weiqiang Zheng

China

Yuhuan Zheng

USA

Weide Zhong

China

Jue-Yu Zhou

China

Lan Zhou

China

Qun Zhou

Vanuatu

Xiaobo Zhou

USA

Xinchun Zhou

USA

Yi-Hong Zhou

USA

Youwen Zhou

Canada

Tao Zhu

China

Yimin Zhu

China

Zhaohui Zhu

China

Guanglei Zhuang

USA

Wenlei Zhuo

China

Volker Ziller

Germany

Daniel Zips

Germany

Inti Zlobec

Switzerland

Amina Zoubeidi

Canada

Valentina Zuco

Italy

Wilbert Zwart

Netherlands

Ellen Zwarthoff

Netherlands

## Pre-publication history

The pre-publication history for this paper can be accessed here:

http://www.biomedcentral.com/1471-2407/14/50/prepub

